# Characterization and Morphology of Nanocomposite Hydrogels with a 3D Network Structure Prepared Using Attapulgite-Enhanced Polyvinyl Alcohol

**DOI:** 10.3390/polym15112535

**Published:** 2023-05-31

**Authors:** Chi-Hui Tsou, Yu-Jie Shui, Juan Du, Wei-Hua Yao, Chin-San Wu, Maw-Cherng Suen, Shuang Chen

**Affiliations:** 1 School of Materials Science and Engineering, Sichuan University of Science and Engineering, Zigong 643000, China; 2 Material Corrosion and Protection Key Laboratory of Sichuan Province, Sichuan University of Science and Engineering, Zigong 643000, China; 3 Department of Materials and Textiles, Asia Eastern University of Science and Technology, New Taipei City 220, Taiwan, China; 4 Department of Applied Cosmetology, KaoYuan University, Kaohsiung County 82101, Taiwan, China; t500088@cc.kyu.edu.tw; 5 Department of Fashion Business Administration, Lee-Ming Institute of Technology, New Taipei City 24305, Taiwan, China

**Keywords:** polyvinyl alcohol (PVA), attapulgite (ATT), nanocomposite hydrogels, nanocomposite xerogel, dye removal efficiency

## Abstract

In this investigation, purified attapulgite (ATT) and polyvinyl alcohol (PVA) were utilized to fabricate nanocomposite hydrogels and a xerogel, with a focus on studying the impact of minor additions of ATT on the properties of the PVA nanocomposite hydrogels and xerogel. The findings demonstrated that at a concentration of 0.75% ATT, the water content and gel fraction of the PVA nanocomposite hydrogel reached their peak. Conversely, the nanocomposite xerogel with 0.75% ATT reduced its swelling and porosity to the minimum. SEM and EDS analyses revealed that when the ATT concentration was at or below 0.5%, nano-sized ATT could be evenly distributed in the PVA nanocomposite xerogel. However, when the concentration of ATT rose to 0.75% or higher, the ATT began to aggregate, resulting in a decrease in porous structure and the disruption of certain 3D porous continuous structures. The XRD analysis further affirmed that at an ATT concentration of 0.75% or higher, a distinct ATT peak emerged in the PVA nanocomposite xerogel. It was observed that as the content of ATT increased, the concavity and convexity of the xerogel surface, as well as the surface roughness, decreased. The results also confirmed that the ATT was evenly distributed in the PVA, and a combination of hydrogen bonds and ether bonds resulted in a more stable gel structure. The tensile properties exhibited that when compared with pure PVA hydrogel, the maximum tensile strength and elongation at break were achieved at an ATT concentration of 0.5%, indicating increases of 23.0% and 11.8%, respectively. The FTIR analysis results showed that the ATT and PVA could generate an ether bond, further confirming that ATT could enhance the PVA properties. The TGA analysis showed that the thermal degradation temperature peaked when the ATT concentration was at 0.5%, providing further evidence that the compactness of the nanocomposite hydrogel and the dispersion of the nanofiller was superior, contributing to a substantial increase in the mechanical properties of the nanocomposite hydrogel. Finally, the dye adsorption results displayed a significant rise in dye removal efficiency for methylene blue with the increase in the ATT concentration. At an ATT concentration of 1%, the removal efficiency rose by 103% compared with that of the pure PVA xerogel.

## 1. Introduction

Hydrogels are a 3D network structure formed by cross-linking hydrophilic polymer materials. It is insoluble in water and it fully swells in water. It can be used in many fields due to its porous and easily expandable properties. Polyvinyl alcohol (PVA) is a hydrophilic material that is simply soluble in an aqueous solution, somewhat soluble in ethanol, but not soluble in most organic solvents [1,2]. It is a good polymer material for preparing hydrogels [3]. PVA hydrogels can commonly be prepared using many kinds of methods, such as chemical methods and physical methods (for example cyclic freezing–thawing method [4]). Compared with others, the physical method of cyclic freezing–thawing to prepare hydrogel has the advantages of high efficiency, convenience and simplicity [5]. The hydrogel prepared using this method will not contain harmful chemicals [6]. However, pure PVA hydrogels generally have limitations, such as low tensile and dye adsorption capability [7,8], and such inadequacies limit their further applications [9]. A better method to enhance the properties of a hydrogel is by enhancing it with nanofillers [10].

Zhang et al. [11] used nanofiller graphene oxide (GO) and PVA to prepare PVA/GO composites hydrogel; when the GO content was 0.7%, the tensile strength was 132% higher than neat PVA hydrogel. To give a hydrogel antibacterial properties, Chen et al. [12] prepared a hydrogel from graphene-doped ZnO and PVA. The results showed that when the graphene-doped ZnO content was 0.3%, the tensile strength improved from 1.9 to 2.2 MPa. A nanocomposite hydrogel also has excellent dye adsorption and antibacterial properties. There are also some studies that investigated carbon nanotubes (CNTs) enhancing the properties of PVA hydrogels [13,14,15]. Tsou et al. [16] used ZnO-decorated carbon nanotubes (CNT-ZnO) to improve the tensile properties of a PVA hydrogel, indicating that when the addition of CNT-ZnO was 0.6%, its tensile strength was significantly increased from 1.1 MPa to 2.3 MPa, and the CNT-ZnO also improved the dye adsorption ability.

Attapulgite (ATT) is a kind of clay mineral found almost all over the world [17]. Because of its unique crystal structure and low price, ATT is widely used in building materials, sewage adsorption treatment [18], agricultural composting and other fields [19,20], with great development prospects [21]. In recent years, it has also received extensive attention from the academic community [22,23]. Compared with other nanomaterials (such as CNT, graphene, and ZnO), ATT is an abundant natural mineral with a low cost. Due to its porous structure, it is more suitable for use as an adsorbent.

Song et al. [24] reported a nanocomposite hydrogel prepared with PVA cellulose and GO, and they added 1, 1.5 and 2% ATT content to enhance the adsorption of Cu and Pb metal ions. The results showed that the adsorption rate of the nanocomposite hydrogel increased with the increase in ATT content. Elbassyoni et al. [25] first modified ATT with hexadecyl trimethyl ammonium chloride and then they added 1, 3, 5, 7 and 10% modified ATT to the PVA hydrogel. The results showed that the tensile strength and breaking elongation of the hydrogel increased with the increase in ATT content, and the swelling degree reached the maximum at 7% ATT content. Farid et al. [26] used the solution casting method to prepare a PVA/carboxymethyl cellulose nanocomposite hydrogel by using citric acid as the crosslinking agent and ATT as a nanofiller (1, 2, 4 and 5%). The results showed that when the ATT content was 1%, the tensile strength and elongation at break of the nanocomposite hydrogel reached the maximum value. This result also indirectly indicated that 1% ATT was not the optimal reinforcement addition. Most related studies showed that ATT can endow hydrogels with mechanical properties and improve their swelling degree and adsorption rate, but so far, the amount of ATT added is more than 1%. A higher ATT content will increase the cost of the nanocomposite hydrogel. Lower nanofillers can not only reduce costs but also avoid agglomeration. Therefore, this study investigated the effect of different low levels of ATT addition on the performance and physicochemical properties of a hydrogel. This new experimental design has the advantage of lower cost compared with other studies and can provide a reference value for future research. The prepared nanocomposite hydrogel was instrumentally characterized using FTIR and SEM, while the tensile property and porosity test of the hydrogel and dye adsorption test of the xerogel were undertaken. The low content of ATT enhanced the tensile strength and durability of the PVA hydrogels through ether bonding and created a nanocomposite hydrogel with a higher thermostability and dye adsorption ability.

## 2. Experimental

### 2.1. Materials

PVA 1799 was used for this study. The purity of the PVA was 99%, the degree of polymerization was 1700 and the degree of alcoholysis was 99%. It was bought from Titan Science Co. Ltd. (Shanghai, China). The purification method used for the ATT was the same as that in a previous work [27]. Deionized water (DI water) was produced using a water machine (ZYTEST, Ulupure, Chendu, China). 

### 2.2. Fabrication of PVA/ATT Nanocomposite Hydrogels

First, PVA pellets were dehydrated in an oven at 85 °C, after which the dried PVA was dissolved in DI water while stirring at 90 °C for 60 min to prepare the PVA solution; the ratio of PVA to water was 15:85. Then, a certain proportion of ATT (0.25, 0.5, 0.75, 1%) was ultrasonicated using a cell disrupter for 5 min. After that, different amounts of ATT were mixed with the PVA solution. Subsequently, the blend was ultrasonicated with a cell disrupter for 12 min and then stirred at 85 °C for 120 min. The blend was transferred into a mold and it was allowed to sit overnight for defoaming. The freezing-and-thawing method was used: the mold and its content were frozen in a freezer set to −20 °C for 24 h, and subsequently thawed in an incubator at 25 °C for 24 h for 5 cycles to obtain PVA/ATT nanocomposite hydrogels, whose components and their amounts are itemized in Table 1.

### 2.3. Water Content

Each test was repeated 4 times, then the average value was calculated. The water content (W_c_) of the PVA/ATT nanocomposite hydrogels was calculated using the following equation:

(1) $$W_{c}=\frac{W_{h}-W_{x}}{W_{h}}\times 100\%$$

where W_h_ is the weight of the hydrogel and W_x_ is the weight of the xerogel.

### 2.4. Swelling Ratio

All samples were put into DI water in an incubator at 25 °C. When the xerogel was fully swollen for 2 days, the water on the surface of the xerogel was removed using filter paper, after which the xerogel was weighed. Each test was undertaken 4 times, then the average value of swelling was calculated. The swelling ratio (*S_r_*) of the composite PVA/ATT nanocomposite xerogel was calculated using the following equation:

(2) $$S_{r}=\frac{W_{s}-W_{d}}{W_{d}}\times 100\%$$

where *W_s_* is the weight of the xerogel after swelling and *W_d_* is the weight of the xerogel.

### 2.5. Gel Fraction

The xerogel was weighed first, then the xerogel was immersed in deionized (DI) water and allowed to fully swell in an incubator at 25 °C for 2 days. Subsequently, it was subjected to vacuum freeze-drying for 2 days before being weighed. Each test was undertaken 4 times, then the average value was calculated. The gel fraction (*G_f_*) of the PVA/ATT nanocomposite xerogel was calculated using the following equation:

(3) $$G_{f}=\frac{W_{f}}{W_{F}}\times 100\%$$

where *W_F_* refers to the weight of the xerogel after the first vacuum freeze-drying process, while *W_F_* refers to the weight of the xerogel after the second freeze-drying process.

### 2.6. Porosity 

The weights of the nanocomposite hydrogel and nanocomposite xerogel were measured using the Archimedean buoyancy method [28]. An electronic balance (BSM-220, Shanghai Zhuojing Electronic Technology, Shanghai, China) was used to determine the weights of all the nanocomposites. Each test was undertaken 3 times, then the average value was calculated. The porosities (*P*) of the PVA/ATT hydrogel and xerogel were calculated using the following equation:

(4) $$P=\frac{W_{2}-W_{1}}{W_{2}-W_{3}}\times 100\%$$

where *W*_1_ refers to the weights of the hydrogel and xerogel in air, *W*_2_ refers to the weights of the nanocomposite hydrogel and nanocomposite xerogel after its complete swelling, and *W*_3_ refers to the weights of the suspended hydrogel and xerogel.

### 2.7. X-ray Diffraction Analysis

XRD (Bruker, Germany) was used on all samples to test the xerogels. The parameters were as follows: Cu-Kα rays, 40 kV voltage, 40 mA current, 2θ angle range from 5–60° and a scanning rate of 0.02°/0.1 s.

### 2.8. Thermogravimetric Analysis

TGA (HTG-1, China) was utilized to conduct thermostability analyses on the xerogels in a nitrogen-protective atmosphere (the flow rate was 70 mL/min, the heating rate was 10 °C/min and the heating range was 25 to 800 °C).

### 2.9. Scanning Electron Microscopy and Energy-Dispersive X-ray Spectroscopy

SEM-EDS (Czech Republic) was utilized to observe the fracture morphologies of the xerogels. Before the xerogels were observed at a magnification of 5 k, the xerogels were gold-plated. The dispersion of ATT in the xerogels was obtained at a magnification of 500 using EDS (QUANTAX, Bruker, Germany).

### 2.10. Surface Profile

The surface morphologies of the PVA and PVA/ATT nanocomposite xerogels after 48 h of freeze-drying and dehydration were tested using a three-dimensional optical profilometer (Contour·GT-K, Tescan, Czech Republic).

### 2.11. Surface Roughness

The surface roughnesses of the dried gels after vacuum freeze drying for 48 h were measured using a surface roughness tester (TR200, DANA, Huzou, China). The sampling length of the equipment was 0.8 mm, each sample was tested 5 times and the average value was calculated.

### 2.12. Water Contact Angle

The surface hydrophilicity of PVA and PVA/ATT nanocomposite xerogels was characterized using an automatically inclined contact-angle-measuring instrument (SDC-350, SINDIN, Chengdu, China). After 48 h of vacuum freeze-drying, the test sample was placed on the testing platform and the syringe automatically dripped 2 μL of deionized water, recorded the contact angle data at 0 s, and the data was automatically fitted and calculated using computer software. Test each was sampled three times and the average value was calculated.

### 2.13. FTIR

An FTIR spectroscopy instrument (Thermo, New York, USA) was utilized to analyze the samples; the test range was 4000–500 cm^−1^ and a resolution of 4 cm^−1^ was set. The FTIR spectra of the following samples were obtained: ATT, pure PVA xerogels and PVA/ATT nanocomposite xerogels.

### 2.14. Tensile Properties

The tests of the tensile properties were carried out using the standard GBT528-2009 microcomputer-controlled universal testing machine (FBS-10KNW, Xiamen Forbes Equipment Co., Ltd., Xiamen, China) at a speed of 50 mm/min and under ambient temperature conditions. Five repetitions of each test were performed, and the average value was computed. The tensile properties of pure PVA, PVA/ATT nanocomposite hydrogels and PVA/ATT nanocomposite xerogels were identified (freeze-drying hydrogels under vacuum conditions for 2 d).

### 2.15. Adsorption Studies

Xerogels (50 mg) of the same sizes were put in 10 mL methylene blue solution (10 mg/L). The shaking speed was 150 rpm a 25 °C for 2 d to reach a state of absorbance equilibrium. The absorbance of the solution was measured using an ultraviolet–visible spectrophotometer (UV-1800PC, Shanghai Jing-Ruo, Shanghai, China) at a wavelength of 664 nm. Each sample was tested 3 times, then the average value was calculated. The removal efficiency (*R_e_*) was calculated using the following equation:

(5) $$Re=\frac{A_{0}-A_{1}}{A_{0}}\times 100\%$$

where *A*_0_ is the initial absorbency of the methylene blue solution and *A*_1_ is the absorbency of the methylene blue solution after the adsorption procedure.

## 3. Results and Discussion

### 3.1. Water Content

**Figure 1:**
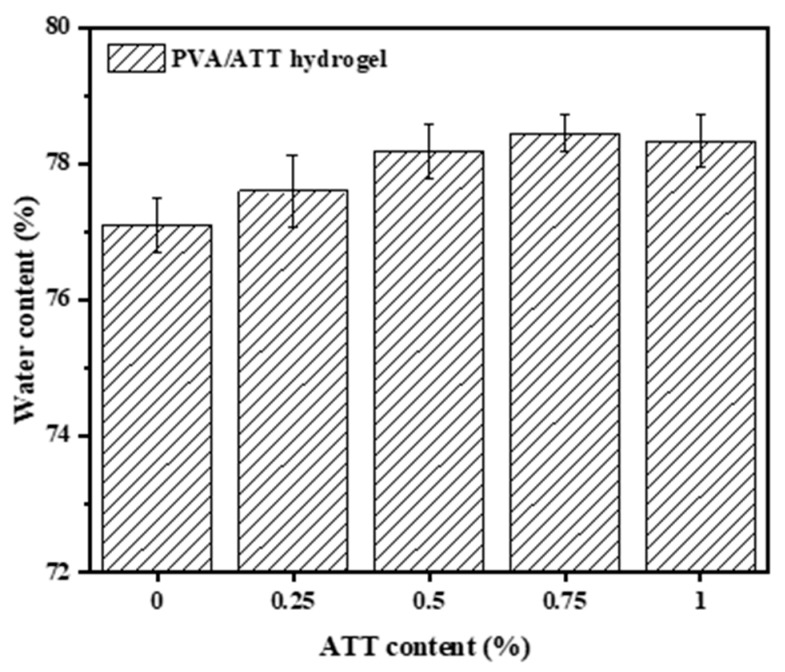
Water contents of the PVA/ATT nanocomposite hydrogels.

Figure 1 discloses the water contents of the hydrogels. The water content was obtained from the weight loss of the hydrogel samples that were freeze-dried for 2 d. The incorporation of ATT had a slight effect on the water content. However, as the amount of the incorporated ATT increased from 0 to 0.75%, the water content also increased. This may have been due to three explanations: First, the amount of water in the hydrogels was the same (Table 1); second, ATT is a porous material, which means it is easily adsorbed inside the porous gel material [29]; third, ATT contains a large number of hydroxyl groups, which is a very hydrophilic nanomaterial [30].

### 3.2. Swelling Ratio and Gel Fraction

**Figure 2:**
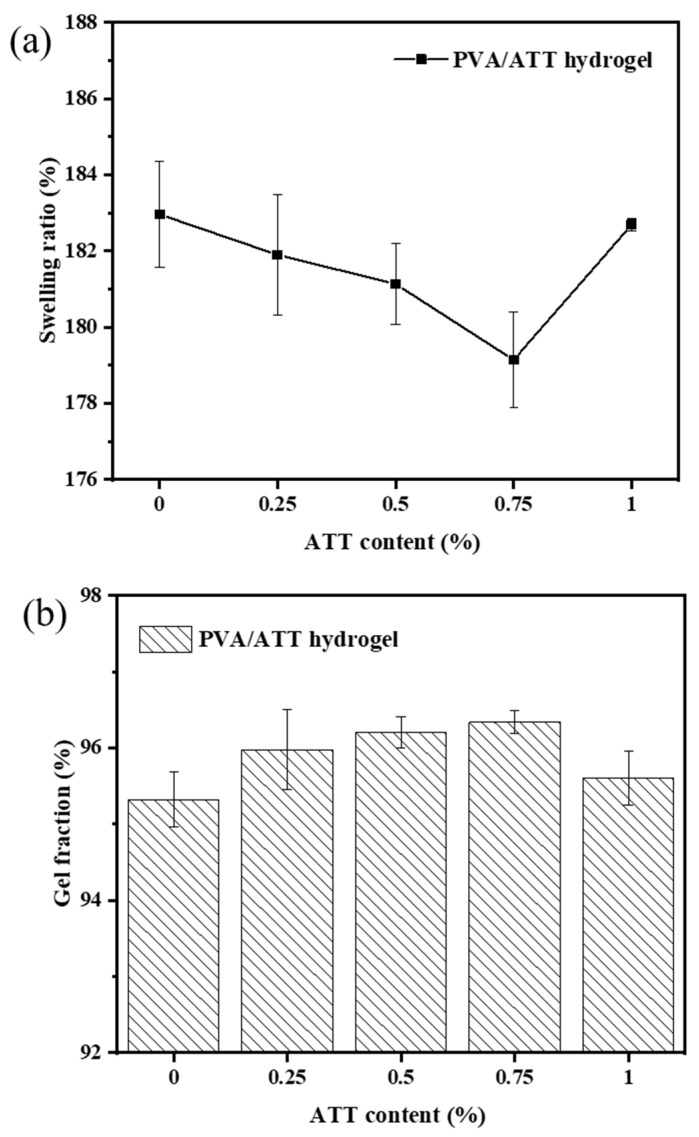
(**a**) Swelling ratio and (**b**) gel fraction for the PVA/ATT nanocomposite xerogels obtained after freeze-drying for 2 d.

Figure 2a shows the swelling ratio for PVA/ATT nanocomposite xerogels that were swollen fully in DI water at 25 °C for 2 d. With the increase in the ATT content, the swelling ratio for the PVA and PVA/ATT nanocomposite xerogels decreased significantly. Because the ATT affected the structure of the xerogel and the blend of the ATT with PVA strengthened the construction between them, this caused an improvement in the nanocomposite xerogel network. The minimum swelling ratio was reached with 0.75% ATT. This effect was similar to that found in the data on hydrogel and xerogel porosity. However, when the content of the ATT nanomaterial reached 1%, the swelling rate rose. This may have been because ATT content = 1% would result in the uneven distribution of nanomaterials, leading to serious agglomeration and an uneven 3D network structure destruction in the nanocomposite hydrogel.

Figure 2b shows the gel fraction of xerogels that underwent a single round of swelling and freeze-drying. The gel fraction could explain the stability between the gel polymer chains. Figure 2b shows an approximately 96% gel fraction for both the PVA and PVA/ATT nanocomposite xerogels. With the increase in the ATT content, the gel fraction of the composite hydrogel showed an upward trend and reached the maximum when the amount of ATT was 1%, which was just opposite to the trend of the swelling coefficient of the gel, which demonstrated that ATT could improve the structural stability of the hydrogel.

### 3.3. Porosity

**Figure 3:**
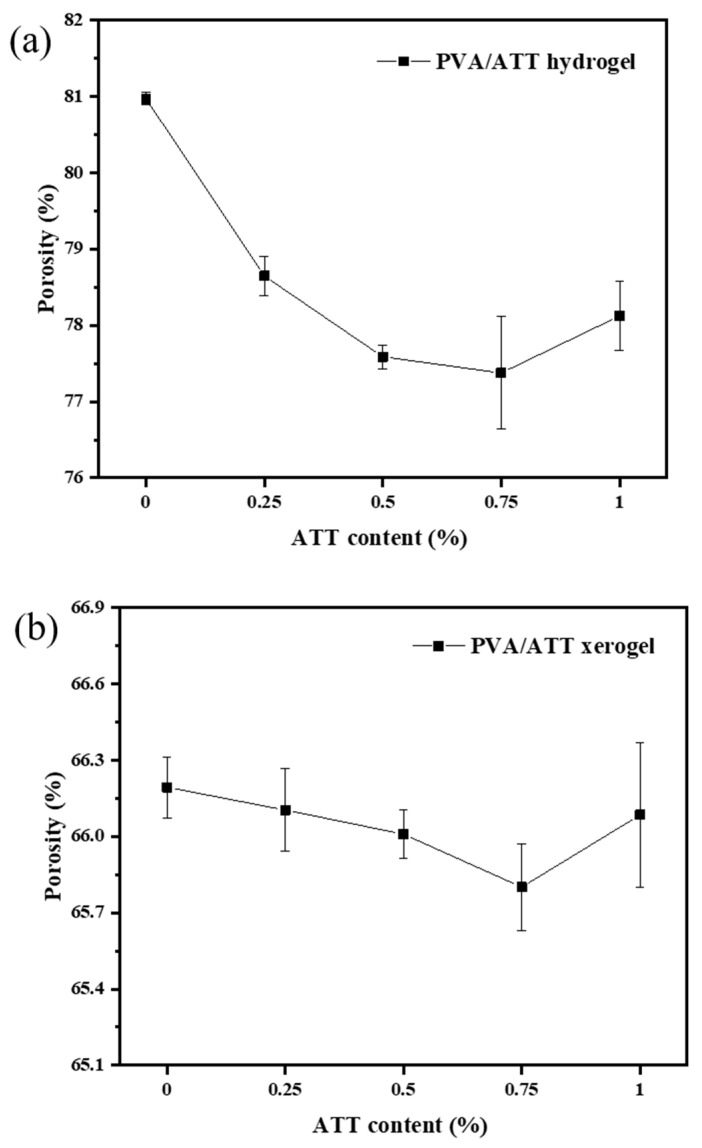
Porosities of the PVA/ATT nanocomposite (**a**) hydrogels and (**b**) xerogels obtained after freeze-drying for 2 d.

The Archimedes buoyancy method was used to measure the porosity of the PVA and PVA/ATT nanocomposite xerogels obtained after freeze-drying the PVA nanocomposite hydrogels and nanocomposite hydrogels for 2 d. Figure 3a,b show the xerogel porosities and hydrogen porosities, respectively. Compared with the porosities of PVA and the PVA/ATT nanocomposite xerogels and hydrogels, the porosities of the PVA and PVA/ATT nanocomposite xerogels were significantly lower than those of the PVA and PVA/ATT nanocomposite hydrogels, which was due to the irrecoverable collapse of some unstable three-dimensional network structures in the gel during freeze-drying, resulting in the lower porosity of the xerogels [31,32].

For the xerogels and hydrogels, the porosity situation shown in Figure 3a,b was similar to that of the ratio of swelling in Figure 2a. In other words, the 3D network structure of the hydrogel had a great correlation with the porosity, and its structure dominated the swelling performance of the composite hydrogel.

### 3.4. X-ray Diffraction

**Figure 4:**
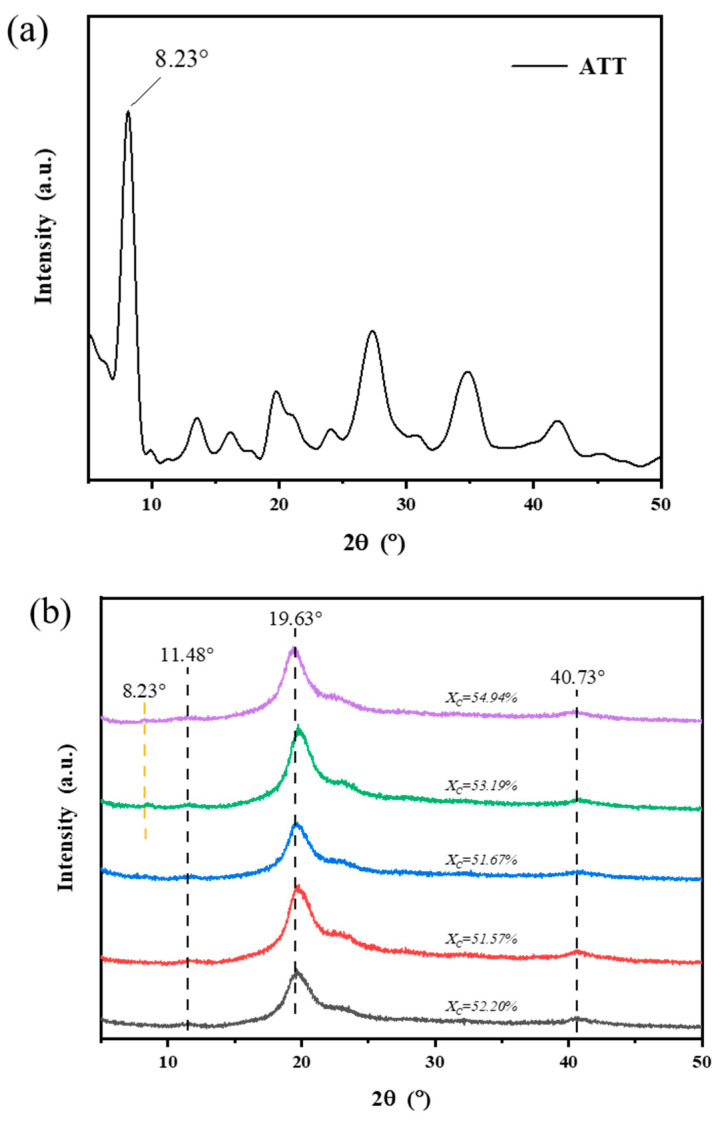
XRD patterns: (**a**) ATT; (**b**) pure PVA and PVA/ATT nanocomposite xerogels.

Figure 4a,b show the XRD diffraction patterns of the ATT and PVA and PVA/ATT nanocomposite hydrogels. Figure 4a shows that ATT had a strong diffraction peak at 2θ = 8.23°, while Figure 4b shows that the obvious diffraction peaks of pure PVA hydrogel were located at 2θ = 11.48, 19.63° and 40.73°, corresponding to the typical PVA crystal phase, and 2θ = 19.63° is also its characteristic peak [33,34].

After the addition of ATT (ATT ≤ 0.5%), the crystallinity of the PVA/ATT nanocomposite xerogels decreased, as shown by the X_c_ values in Figure 4b. When the ATT contents were 0.25% and 0.5%, the crystallinities were very low and close to each other, which indicated that the nanofillers in these xerogel samples were consistently dispersed. The nucleation was excellent and the crystal size was smaller. When the ATT content ≥ 0.5% (0.75 and 1%), the crystallinity increased, which may have been due to the slightly poor dispersion. On the other hand, it can be observed that when the ATT content reached 0.75 and 1% in the PVA/ATT nanocomposite xerogels, a new peak at 2θ = 8.23° suddenly appeared. This peak was the same as the characteristic peak of ATT (see Figure 4a). This may be attributed to the high content of ATT or partial agglomeration on the surface, meaning that XRD detect could detect it easier.

### 3.5. Thermogravimetric Analysis (TGA) and Differential Thermogravimetry (DTG)

**Figure 5:**
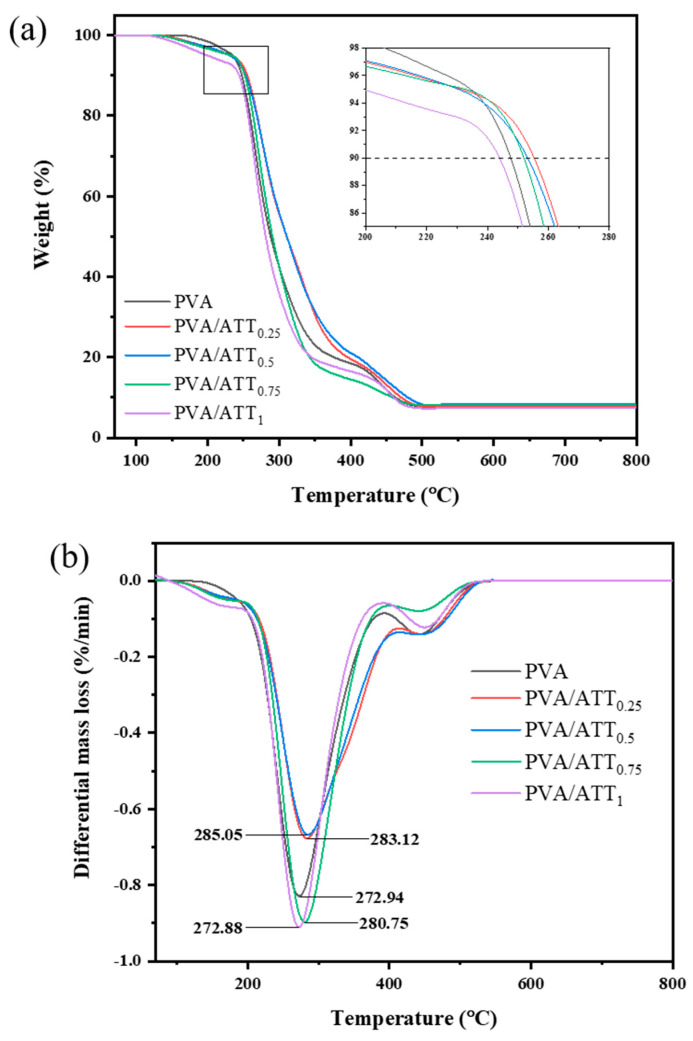
(**a**) TGA and (**b**) DTG curves: pure PVA and PVA/ATT nanocomposite xerogels.

Figure 5a,b show the TGA and DTG curves for the neat PVA and PVA/ATT nanocomposite xerogels. It can be seen that the thermodegradation of the PVA and PVA/ATT nanocomposite xerogels all had two weight loss stages. The temperature in the first step was about 190–310 °C, which involved the degradation of PVA side chains [35]. The first stage was the fastest stage of thermodegradation of the nanocomposites. The second step occurred at temperatures of approximately 300–450 °C, which was connected to C-C backbone cleavage in PVA polymer [36].

Table 2 lists the detailed data for TGA and DTG. T_1_ values for TGA of the PVA/ATT nanocomposite xerogels were similar to that of pure PVA hydrogel, whereas T_2_ values for TGA were higher than that of PVA when the ATT content was 0.25–0.5%, and T_2_ was the highest at 0.5% ATT. It can be also observed that DTG peak 1 shows that the 0.5% ATT content reached the maximum values. This indicated that a small amount of ATT (0.25–0.5%) could improve the thermostability of the nanocomposite xerogel.

### 3.6. Morphology Analysis

**Figure 6:**
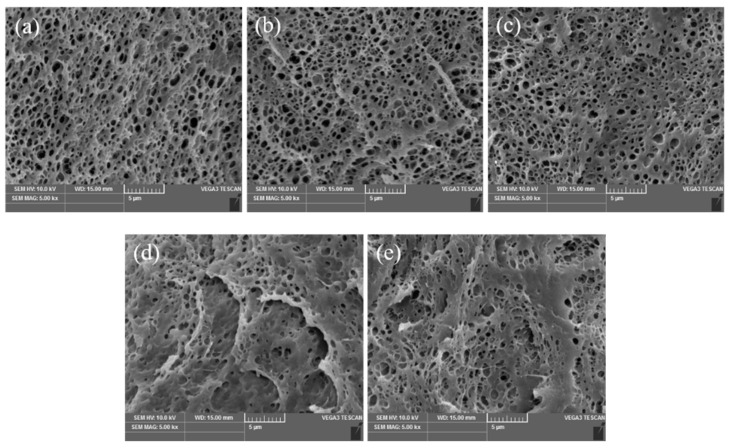
SEM images: (**a**) pure PVA; (**b**) PVA/ATT_0.25_; (**c**) PVA/ATT_0.5_; (**d**) PVA/ATT_0.75_; (**e**) PVA/ATT_1_.

Figure 6 shows the fraction section morphology of the pure PVA and PVA/ATT nanocomposite xerogels. From Figure 6a, it can be observed that the section of the pure PVA xerogel had several irregular pores with many different sizes. The surface of the nanocomposite xerogels did not change much with the addition of ATT from 0.25 to 0.5%. It can be seen from Figure 6b,c that with the increase in the amount of added ATT nanomaterials, the holes of the structure of the PVA/ATT nanocomposite xerogels were similar looking, with only slightly smaller pores, but this was not obvious. This may be attributed to the fact that ATT dispersed well in the PVA xerogels, as well as forming ether bonds and hydrogen bonds with the PVA, making the structure denser. When the added amount of ATT ≥ 0.75% (see Figure 6d,e), it may have been that a small amount of nanofiller was agglomerated, and thus, the uneven dispersion of ATT led to the partial collapse of the uniform porous continuous structure and wrinkles. Thus, the porosity and swelling degree reached the lowest value (see Figure 2a and Figure 3a,b).

### 3.7. EDS Analysis

**Figure 7:**
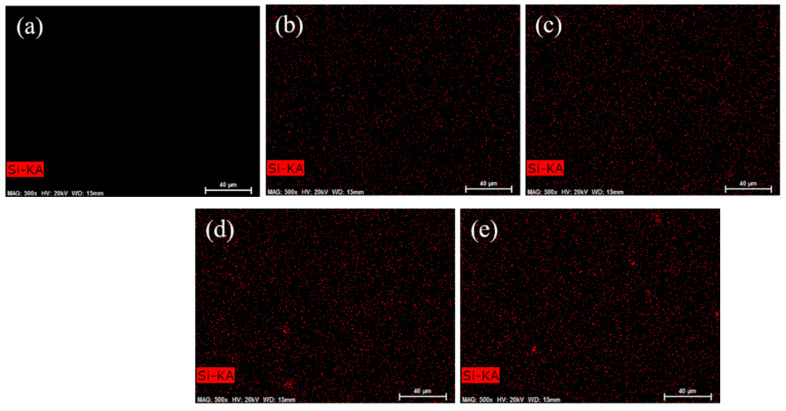
EDS images: (**a**) neat PVA; (**b**) PVA/ATT_0.25_; (**c**) PVA/ATT_0.5_; (**d**) PVA/ATT_0.75_; (**e**) PVA/ATT_1_.

To further confirm the dispersion of ATT in PVA, EDS analysis results are shown in Figure 7. Figure 7 displays the dispersal of Si in the neat PVA and PVA/ATT nanocomposite hydrogels. Figure 7a confirms that the pure PVA hydrogel did not contain Si. Figure 7b–f illustrate the distribution of Si in PVA/ATT nanocomposite hydrogels (ATT content: 0.25–1%). From the EDS analysis, when the amount of ATT was less than 0.75% (0.25 and 0.5%), ATT was evenly distributed in the PVA xerogel. When the ATT content reached 0.75% and above, there were some agglomeration phenomena. This was consistent with the SEM characterization results. When the doping amount was higher than 0.5%, the three-dimensional network in the hydrogel was slightly damaged. The ATT part filled the porous structure, reducing the holes and making the surface smoother. Consequently, there was no further enhancement in the mechanical properties at more than 0.5% ATT. With the addition of more ATT, the section’s pore structure became lower. The reduction in the number of pores led to difficulty of the entrance of H_2_O molecules, resulting in the reduced swelling ratio of the xerogels.

### 3.8. Surface Profile

**Figure 8:**
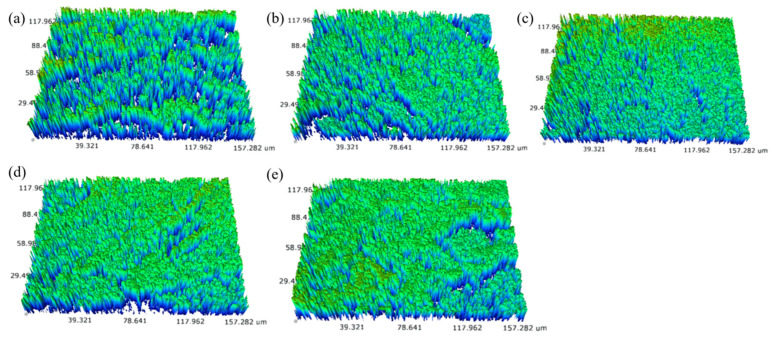
Surface profile images: (**a**) pure PVA; (**b**) PVA/ATT_0.25_; (**c**) PVA/ATT_0.5_; (**d**) PVA/ATT_0.75_; (**e**) PVA/ATT_1_.

Figure 8 shows the surface profile 3D images of the PVA and PVA/ATT nanocomposite xerogels tested using a 3D optical profilometer. It can be seen from the figure that the surface of the pure PVA dry gel was uneven, with obvious ups and downs, and there was a dense deep pore structure. This was because the hydrogel had a three-dimensional network and porous structure. With the loss of water in the hydrogel during dehydration, the intermolecular force of the PVA was enhanced and the three-dimensional network structure shrank and collapsed unevenly, and the gel surface appeared more uneven. With the increase in ATT content, the concavity and convexity of the dry gel surface decreased, and the deep pore structure decreased and gradually became flat, which was consistent with the roughness test results. This may have been because the ATT was evenly dispersed in the PVA dry gel and connected with the PVA by hydrogen bonds and ether bonds. The structure of the gel was more stable and the structure of the wet gel was more complete during dehydration. It can be clearly seen from Figure 8d,e that when the amount of ATT added was ≥0.75%, the concavity and convexity of the dry gel surface were lower than that before the addition of the ATT, but there were obvious cracks. At this time, there was a weak point of stress in the composite, which may have been one of the reasons for the reduction in the mechanical strength of the dry gel [37]. The cracks may have been caused by the agglomeration of the ATT, which destroyed the continuity of the structure in the gel, which was mutually confirmed by the SEM test results of the liquid-nitrogen-quenching section of the hydrogel.

### 3.9. Surface Roughness

**Figure 9:**
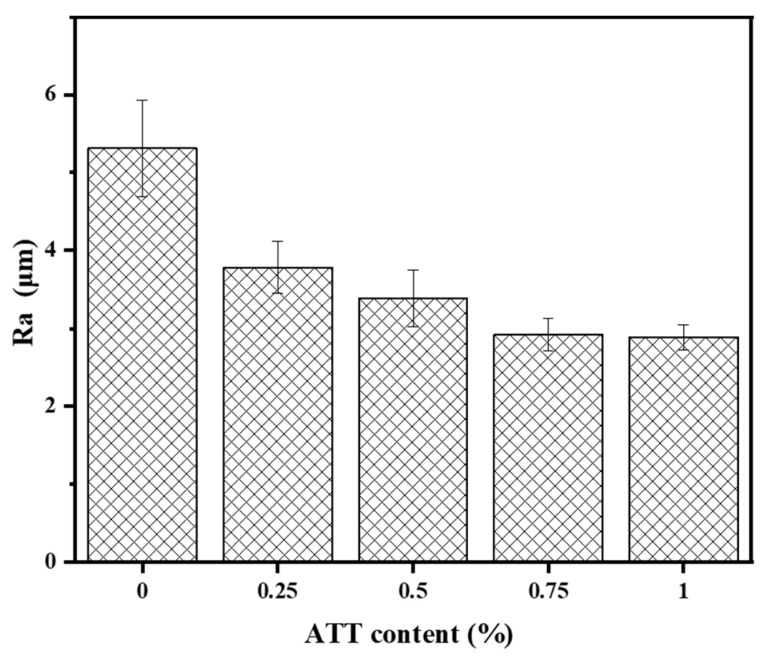
Surface roughness of the PVA and PVA/ATT nanocomposite xerogels.

Figure 9 shows the roughnesses of the nano PVA and PVA/ATT nanocomposite xerogels with different contents after 48 h of freeze-drying and dehydration. From the figure, it can be seen that as the amount of ATT added increased, the surface roughness of the composite material decreased, and there was basically no change from 0.75% to the lowest value. The reason for this result may be that the ATT nanomaterials were evenly distributed in the wet PVA and covered the pores of the dry gel, making the surface of the dry gel smoother [38], which was consistent with the porosity test and SEM observation results of the hydrogel. At the same time, ATT contains multi-hydroxyl functional groups, which can form hydrogen bonds or react with the hydroxyl groups of PVA to form ether bonds, which makes the three-dimensional network structural connections of the composite dry gel more compact and more stable, resulting in a decline in surface roughness [39].

### 3.10. Water Contact Angle

**Figure 10:**
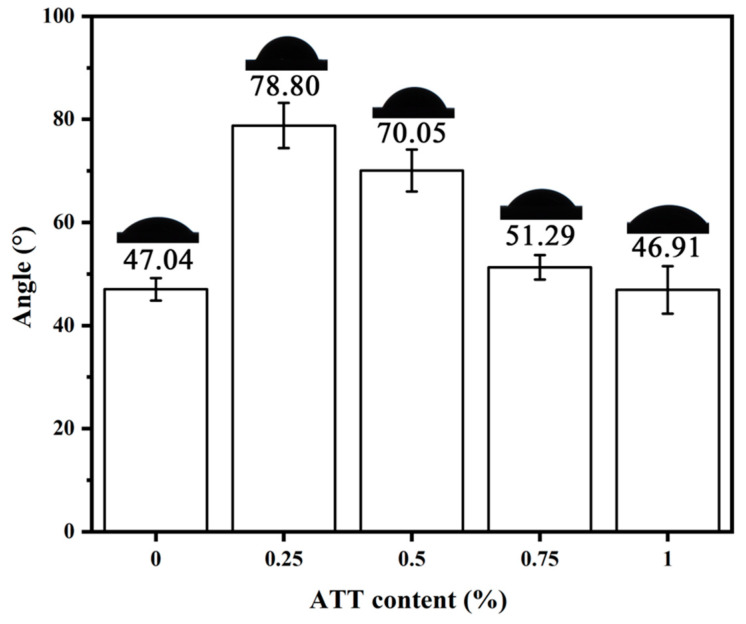
Water contact angles of the PVA and PVA/ATT nanocomposite xerogels.

Figure 10 shows the water contact angle of the PVA and PVA/ATT nanocomposite xerogels at 0 s after 48 h freeze-drying and dehydration. From the graph, it can be observed that the contact angle of the pure PVA was about 47.04°, indicating that the PVA was a hydrophilic material [40]. However, the contact angle of the PVA/ATT composite materials showed a trend of first increasing and then decreasing after the addition of the ATT. When the ATT content was 0.25%, the water contact angle of the PVA and PVA/ATT nanocomposite xerogels reached its maximum value of 78.80°. With the further increase of ATT, its contact angle value became lower and lower. When the content was 1%, the contact angle reached the minimum value of 46.91°, which was very close to the value of the pure PVA contact angle. The reason for the increase in the water contact angle was that the addition of ATT made the PVA/ATT nanocomposite xerogels connections dense, and the instantaneous contact angle increased when water contacted the xerogels; the results of surface roughness analysis showed that the surface roughness of the dry gel was reduced by adding ATT. According to Wenzel’s research conclusion [41], when the roughness of hydrophilic materials decreases, their water contact angle will be larger. Subsequently, as the content of the ATT increased, the water contact angle showed a decreasing trend. Although the surface roughness can affect the value of the contact angle due to the large number of hydrophilic hydroxyl groups and the porous structure that can easily absorb water on the surface of the ATT, the PVA/ATT nanocomposite xerogels showed an increasingly hydrophilic trend as the amount of ATT added increased.

### 3.11. FTIR Spectroscopy

**Figure 11:**
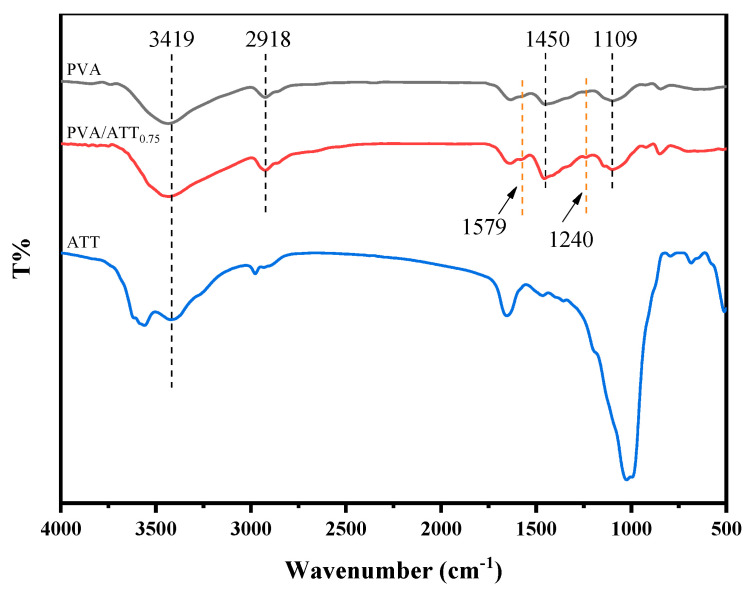
FTIR spectra of the ATT, pure PVA and composite PVA/ATT_0.75_ nanocomposite xerogel.

Figure 11 shows the infrared spectra of the ATT, PVA, and PVA/ATT nanocomposite xerogels. PVA showed several characteristic bands. The FTIR spectrum of the ATT in Figure 11 showed that there was a clear absorption peak at 3419 cm^−1^, which was the OH vibration peak. The peak at 2918 cm^−1^ was the symmetrical stretching vibration of C-H. The peak at 1450 cm^−1^ was the C-OH stretching vibration peak, and the peak at 1109 cm^−1^ was related to the stretching vibration of the C-O group. When the ATT was added to the PVA xerogel, its PVA/ATT nanocomposite xerogel showed two more obvious peaks at 1240 cm^−1^ and 1579 cm^−1^. This may be attributed to the ether bond formed between the O-H group of PVA and the O-H group of ATT [42].

**Figure 12:**
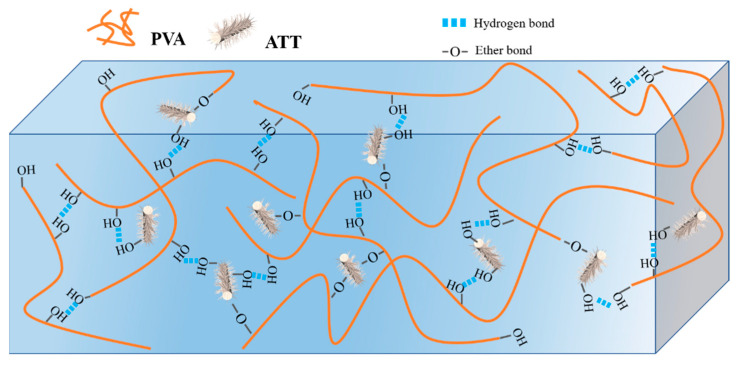
Structure of the nanocomposite PVA/ATT hydrogel.

The possible reaction mechanism for the network structure formed between the PVA xerogel and nanomaterial ATT is illustrated in Figure 12, which describes the ether bond with the PVA matrix or the hydrogen bonds between the PVA matrix and the ATT nanofiller.

### 3.12. Mechanical Properties

**Figure 13:**
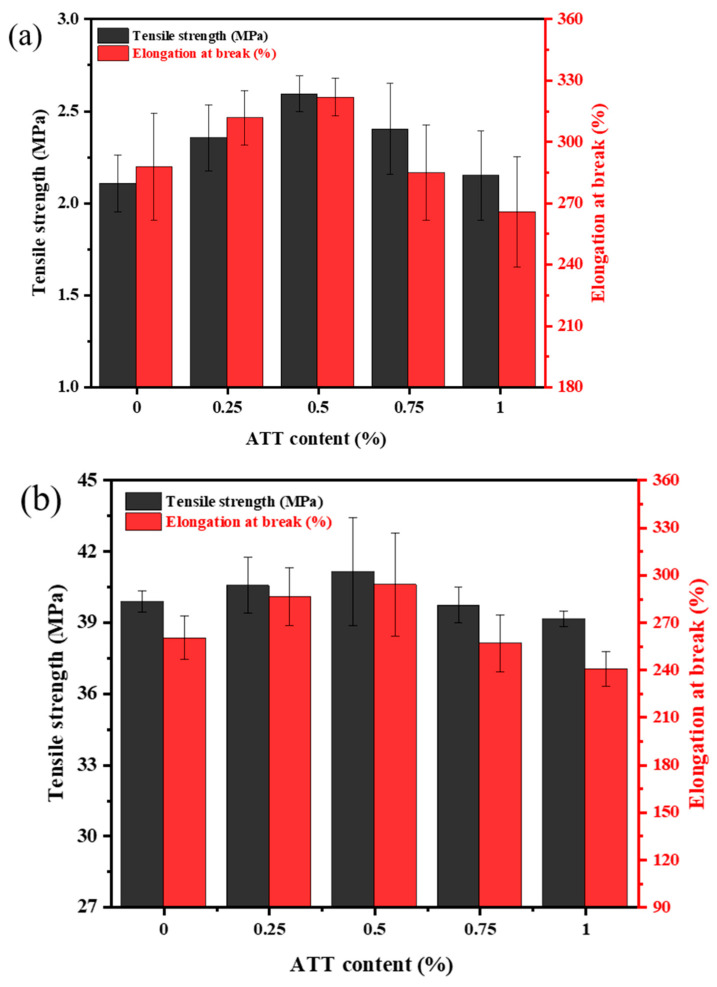
(**a**) Mechanical properties of the PVA/ATT nanocomposite hydrogels. (**b**) Mechanical properties of the PVA/ATT nanocomposite xerogels.

Figure 13a displays the tensile properties of the PVA/ATT nanocomposite hydrogels. As the amount of the added ATT increased from 0 to 0.5%, both the tensile strength and elongation at break greatly increased. The maximum tensile strength of 2.59 MPa and elongation at break of 322% were reached when the ATT was 0.5%. This might be attributed to ATT nanofiller having a good effect on promoting the properties of the polymer matrix [43]. In addition, the hydroxy (-OH) of ATT also formed hydrogen and ether bonding with the hydroxyl group from the PVA, and this promoted the improvement of the mechanical properties of the nanocomposite hydrogels [44].

Nevertheless, the tensile strength and elongation at break were reduced at amounts of ATT greater than 0.5%. The decrease in tensile strength of the nanocomposite hydrogels when the incorporated amounts of ATT were 0.75 and 1% may have been due to the uneven dispersion of ATT nanofiller in the hydrogel, causing the 3D network structure of the hydrogels to become damaged. This was consistent with the trend phenomenon shown in the SEM characterization (see Figure 6), which showed a reduction in its porous structure.

Figure 13b shows the tensile strength and elongation at break of the xerogels. The pure PVA xerogel in Figure 10b indicated a tensile strength of 39 MPa. In the case of 48 h freeze-drying at an ATT addition of 0.5%, the tensile strength of the nanocomposite xerogel, as shown in Figure 10b, is clearly greater by 16 times than that of the hydrogel samples, as indicated in Figure 10a. This may have been due to the xerogel releasing a lot of water, and thus, more hydroxyl groups from the samples. As such, the interaction between the hydroxyl groups formed tougher intramolecular hydrogen bonds [45,46]. Consequently, the tensile properties of the PVA and PVA/ATT nanocomposite xerogels were obviously enhanced compared with the hydrogels sample [47,48]. The trend of the tensile strength and elongation at break for the xerogels is shown in Figure 13b. The tensile strength increased with the nanofiller content; at 0.5% ATT, it reached approximately 41 MPa with 48 h freeze drying. Nevertheless, when the ATT was 0.75%, the tensile strength and elongation at break decreased, perhaps because of the agglomeration of the ATT [49,50]. When the addition amount ranged from 0.75% to 1%, the mechanical properties further declined, which was consistent with the EDS characterization results (Figure 7d,e) showing that the agglomeration phenomenon became more serious [27].

### 3.13. Dye Adsorption Studies

**Figure 14:**
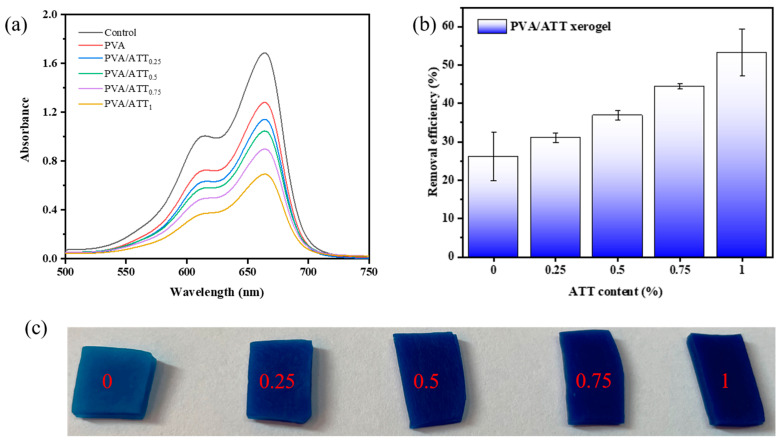
(**a**) Adsorbance of methylene blue solution by nanocomposite hydrogels in the equilibrium state. (**b**) Effect of ATT content on the removal efficiency of methylene blue solution. (**c**) Image after 48 h of adsorption onto the xerogel.

Figure 14a,b show the methylene blue solution absorbance and the removal efficiency, respectively. Figure 14c shows the image of the xerogel after 48 h of adsorption. The following samples in the amount of 0.05 g were added in 10 mL methylene blue solution (10 mg/L) at 25 °C for 2 d: a blank control, neat PVA sample and PVA/ATT nanocomposite xerogels obtained after freeze-drying hydrogels in a vacuum for 2 d.

As can be discerned from Figure 14b, with the increase in the amount of ATT incorporated in the xerogel samples, the adsorption capacity of methylene blue solution on the PVA/ATT nanocomposite xerogels increased. The dye adsorption effect of this nanocomposite hydrogel was significantly better than that of the other two studies [12,16]. The main reason may have been that ATT is a porous material [27], which can provide more binding points with methylene blue, and thus, more effectively adsorb dyes.

## 4. Conclusions

This research provided significant insights into the role of purified attapulgite (ATT) in enhancing the properties of PVA nanocomposite hydrogels and xerogel. The key findings confirmed that the inclusion of 0.75% ATT led to a peak in the water content and gel fraction of the PVA nanocomposite hydrogel. Simultaneously, this ATT concentration contributes to minimized swelling and porosity of the nanocomposite xerogel. Further analysis using SEM, EDS and XRD methods established that an ATT concentration of 0.5% allowed for even dispersion of the nanosized ATT, thereby optimizing the porous structure. Beyond this level, the ATT started to aggregate, impairing the porous structure. Notably, the appearance of a distinct ATT peak at concentrations of 0.75% and above confirmed the agglomeration effect. The research also found that the optimal tensile strength and elongation at break were achieved at an ATT concentration of 0.5%, leading to substantial improvements of 23.0% and 11.8%, respectively, compared with pure PVA hydrogel. The existence of an ether bond, as observed using FTIR analysis, between ATT and PVA suggests the enhancement of PVA properties by ATT. Significantly, an ATT concentration of 0.5% led to a peak thermal degradation temperature, corroborating the superior compactness of the nanocomposite hydrogel and the excellent dispersion of the nanofiller. An increase in the ATT content led to a decrease in the surface roughness and porosity, which was attributed to the even dispersion of ATT and its binding with PVA through hydrogen and ether bonds. The increase in ATT content initially increased the water contact angle, making the material less hydrophilic, but this effect was mitigated with further increases in ATT content. Moreover, the ATT concentration increase was found to drastically enhance the dye adsorption capacity, with a remarkable 103% increase in the removal efficiency at 1% ATT concentration compared with the pure PVA xerogel. In conclusion, the incorporation of ATT at the appropriate concentration significantly improved the properties of PVA nanocomposite hydrogels and xerogel, including the mechanical and thermal properties, as well as the dye removal efficiency. These findings underscore the potential of this enhanced nanocomposite hydrogel for diverse applications in areas such as medical or environmentally friendly materials. Further research can explore other potential advantages and implications of ATT in this context, thereby broadening its scope of application.

## Figures and Tables

**Table 1:** Make-up of the PVA/ATT nanocomposite hydrogels.

Sample	PVA (%)	ATT (%)
PVA	100.00	0
PVA/ATT_0.25_	99.75	0.25
PVA/ATT_0.5_	99.50	0.50
PVA/ATT_0.75_	99.25	0.75
PVA/ATT_1_	99.00	1

**Table 2:** Thermogravimetric analysis/differential thermogravimetry of PVA/ATT nanocomposite xerogels.

Samples	*T*_1_ (°C)	*T*_2_ (°C)	DTG Peak 1 (°C)	DTG Peak 2 (°C)
PVA	247.6	358.4	272.9	438.4
PVA/ATT_0.25_	255.3	382.7	283.1	442.9
PVA/ATT_0.5_	253.1	393.8	285.0	445.4
PVA/ATT_0.75_	251.9	336.6	280.7	445.4
PVA/ATT_1_	243.8	335.6	272.9	449.2

## Data Availability

Data sharing is not applicable.
